# Understanding the impact of different hand drying methods on viral aerosols formation and surface contamination in indoor environments

**DOI:** 10.3389/fpubh.2025.1664322

**Published:** 2025-10-22

**Authors:** Ines B. Moura, Karen Bentley, Kimrun Kaur, Mark H. Wilcox

**Affiliations:** 1 Leeds Institute of Medical Research, Faculty of Medicine and Health, University of Leeds, Leeds, United Kingdom; 2 Department of Microbiology, Leeds Teaching Hospitals NHS Trust, Leeds, United Kingdom

**Keywords:** hand drying, paper towels, electric hand dryer, viral contamination, aerosols

## Abstract

**Background:**

As COVID-19 restrictions were lifted, compliance with good hygiene practices has declined. Hand drying can help remove microbes that remain on hands following poor hand washing. We looked at the potential of new electric hand dryer models to disperse microbial droplets and aerosolized particles during hand drying, to understand if there is a potential infection risk.

**Methods:**

We used both a food dye solution and a bacteriophage solution to visually and quantitively investigate the potential of electric hand dryers Airblade 9KJ (A9KJ), Airblade Wash & Dry (AW+D) and of paper towels (PT), to disperse water droplets in the washroom environment, potentially contaminating surfaces, the user, and a bystander. We also investigated whether microorganisms aerosolized during hand drying can contaminate facemasks of others sharing the same space, mimicking the risk of virus inhalation, up to 30 min post-hand drying.

**Results:**

The highest level of droplet contamination on the floor and walls was observed using the A9KJ hand dryer. Compared to PT, average wall contamination was 78 times higher with A9KJ, and 19 times higher with AW+D. Hand drying assays using bacteriophage showed significantly less splattering contamination of both masks and torso when using PT, compared with electric hand dryers’ use. Overall, person contamination was 100- to 1,000-fold lower at the hand dryer position when using PT. Mask contamination of participants standing at 1 m distance of the hand drying unit was 10-fold and 100-fold lower in assays using PT, compared to A9KJ hand dryer and AW+D wall hand dryer use, respectively.

**Conclusion:**

The potential for virus spread via droplets and aerosols was considerably higher following the use of electric hand dryers, suggesting users are more at risk of contact with viral particles via touching contaminated surfaces or inhalation when using electric hand dryers, compared with PT.

## Introduction

Hand drying is an important step of hand hygiene, that complements hand washing by assisting with the removal of microbial contamination from hands (1). This step became additionally important during the COVID-19 pandemic, as appropriate hand washing practices increased with the public awareness to their role in reducing the virus spreading through contaminated surfaces (2–4). However, the end of social restrictions has been accompanied by a decline in compliance with good hand hygiene practices (3, 4).

This change in behavior away from recommended guidelines (5) can have public health implications. Respiratory infections caused by SARS-CoV-2, influenza and other respiratory virus have been rising in Western countries since 2021 (6–9), leading to variable uptake of facemask use, notably for enhanced protection of those more susceptible to severe disease (10, 11). However, as influenza and SARS-CoV-2 virus can also survive on contaminated hands and surfaces in community settings (12, 13), there is an increased risk of viral spread in contaminated environments, namely hospitals, that are often used by susceptible individuals.

Previous studies have shown PT are more effective at removing moisture and pathogens from hands (1, 14–17), compared to the jet air dryer or warm air dryer models. PT were also associated with a lower potential to disperse droplets (18–22) and lower particle aerosolization (20, 22). Jet air dryers have been associated with droplet dispersion up to 1.5 m for viral particles (21) and over 3 m for bacterial particles (23). These results demonstrate the potential risk of airborne dissemination of microbial pathogens during and following hand drying, according to the method used.

**Figure 1:**
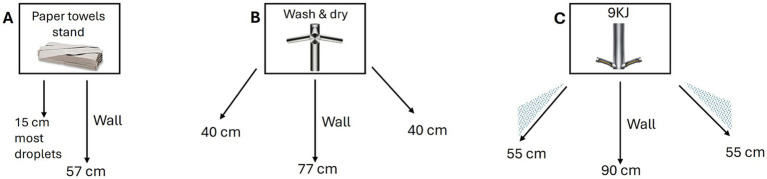
Schematics of droplet dispersion observed with each hand drying method. Arrows represent the widest angle and longest distance traveled by droplets following hand drying with **(A)** paper towels, **(B)** Dyson AW+D wall hand dryer, **(C)** Dyson A9KJ hand dryer.

New high-speed electric hand drying systems (Figure 1A) have become available, including some that combine hand washing and drying (Figure 1B) in a small footprint and are commonly found in high traffic public toilets such as in airports, hospitals, and train stations. The potential of these systems to disseminate droplets or aerosolized particles that can remain in the environment and contaminate others for an extended period after hand drying remains poorly studied. Therefore, using a bacteriophage as surrogate for hand microbial contamination we investigated: (i) whether different hand drying methods impacted the residual microbial contamination remaining on hands following hand drying of poorly washed hands and, (ii) the potential of each hand drying method to promote aerosolization of bacteriophage particles that can contaminate standby users’ masks (as a proxy of inhalation risk) during and following hand drying.

## Materials and methods

### Food dye assays to assess washroom contamination following hand drying

A total of 3 hand drying methods were investigated: Dyson Airblade 9 kJ (A9KJ) hand dryer, the Dyson Airblade Wash+Dry (AW+D) wall dryer, and paper towels (PT). Both the A9KJ and AW+D dryer have a 2-arm design and are activated by placing the hands beneath the dryer arms and moving them parallel to the dryer surface. However, with the A9KJ hands are dried at a 30-degree flexion with an outlet air velocity of 158 m/s, whereas using the AW+D wall dryer, hands are extended horizontally and dried with an air velocity of 153 m/s.

For each method, 3 hand drying assays were performed to assess droplet dispersion within the washroom environment. A power calculation was not performed as the purpose of these experiments was to visually assess the droplet contamination caused by each method and to inform the surfaces to be tested in the subsequence bacteriophage assays.

Hand dryer users immersed their hands in 10 mL of food dye solution and performed hand drying following manufacturer recommendations for each method, i.e., 10s with A9KJ hand dryer, 14 s with Dyson AW+D wall hand dryer, or using 3 PT.

All experiments were performed in presence of a standby user stationed at ~1 m distance from the hand drying station.

During hand drying, all participants wore personal protective equipment including a disposable Tyvek protective suit (DuPont, Stevenage, United Kingdom) and face shields (Fisher Scientific, UK) for visual assessment of user contamination. Furthermore, selected areas were monitored to determine the level of droplet dispersion caused by each hand drying method, namely: (i) wall and floor sections of 65 cm x 40 cm both underneath the dryer and at 1 m distance, corresponding to the width and length of space occupied by an average person while standing still, (ii) a visor shield surface of 32 cm x 22 cm, representing the face area exposed to droplets, (iii) Tyvek suit torso and leg areas of 16 cm x 22 cm, selected based on the shortest hand drying volunteer. Splattering was measured by counting droplets over 1 mm in diameter in the sectioned areas. For each assay, droplets were counted manually by 2 people using a digitally counter pen, and the average value was recorded.

### Power calculation for bacteriophage assays

The study was powered to detect a difference between two methods, based on the contamination remaining on hands following hand drying and then transferred to a surface (door handle). The calculation was based on the risk of microbial dissemination to surfaces, associated with poorly cleaned hands, as previously reported (1). Experimental data was used to inform the calculation (1). Assuming non-normally distributed data with a population standard deviation of 22,347 between the two arms and analyzed using a Mann–Whitney test, it was determined that at least 6 experiments per arm would be required to find a difference of 10^3^ copies/μL (or 10-fold), with 90% power, and alpha error rate of 0.05%.^1^ As different hand dryer models were used in this experiment, we have performed 10 assays per arm instead of the 6 deemed essential. This allowed us to also investigate standby user contamination during hand drying, and mask contamination via droplets and aerosolized particles, in addition to hand and surface contamination.

### Preparation of bacteriophage filtrate

Bacteriophage PR772 (BAA-769-B1) was propagated using its recommended host strain *Escherichia coli* K12 (BAA-769), as previously described (1). The obtained bacteriophage filtrate was diluted to 10^8^ pfu/ml and kept at 4 °C until use.

### Bacteriophage dispersion and aerosol formation during hand drying

All hand drying tests were carried out in a room measuring 48 m^3^ without air-conditioning at the Leeds General Infirmary (United Kingdom) (20). Room air was renewed through standard ventilation, without applied active positive or negative pressure (i.e., no fans or air conditioning). Before each experiment, all surfaces were sanitized with chlorine wipes (Medipal, Pal, United Kingdom). Before each test, volunteers sanitized their gloved hands with 70% alcohol hand gel disinfectant (Sterillium, Germany), followed by immersion in 10 mL of bacteriophage solution. Hands were shaken thrice to remove excess liquid and dried using either the electric hand dryers A9KJ (Dyson, United Kingdom) for 10s, the Dyson AW+D wall hand dryer (Dyson, United Kingdom) for 14 s, or using three PT (Hand Towels H3, Tork, United Kingdom).

Overall, each method was tested in 10 separate hand drying assays performed on 2 different days (5 assays per day). Surface and mask samples were collected after each individual assay; however, the 5 daily experiments were performed consecutively, without additional ventilation of the room between assays. This aimed to replicate the potential contamination occurring following repeated use of public washrooms. All assays were performed in presence of a standby user stationed at 1 m and at a 45-degree angle from the hand drying station.

During hand drying, each participant wore a N95 respirator (FFP2NR, Omnitex) and a disposable plastic apron (Medisave UK Ltd., United Kingdom), for measurement of body/clothing contamination. Once hand drying was completed, one of the hands of the volunteer performing the hand drying was immediately sampled (palm and fingertips) to measure contamination remaining on hands after drying. With the other gloved hand, the volunteers touched a door handle as they would for standard use. All surfaces (hands, apron, gloves) were swabbed with a 3 M sponge-stick moistened with neutralizing buffer (SLS, United Kingdom). Surfaces were disinfected with chlorine wipes pre- and post-sampling, as previously described (1).

Facemasks worn during hand drying were collected to measure mask contamination occurring as result of splattering. All volunteers then wore a new respirator and remained at their positions for 5 min, allowing any potential aerosols to settle on the clean masks. This process was performed twice at 10–15 min post-hand drying, and at 25–30 min post-hand drying, to assess if aerosolized particles could potentially deposit on the masks and be detected by qPCR analysis, as previously reported (22).

### Bacteriophage recovery from masks

All N95 respirators were individually bagged upon collection and transferred to the lab for immediate testing. The outer layer of each mask was removed using sterile scissors and saturated with 2 mL of DNA/RNA shield solution (Zymo Research, Germany), as described before (24). The elute was recovered via centrifugation for 1 min at 3,300 g and stored at 4 °C until DNA extraction.

### DNA extraction and quantitative PCR (qPCR)

DNA extraction was performed using the QIAamp 96 Virus QIAcube HT Kit and 400 μL of elutes from masks or surfaces (sampled with 3 M sponge-stick). Bacteriophage quantification was performed as previously described (1) via qPCR and using primers specific for gene P3 of bacteriophage PR772 (Table 1). Standard curves were used to convert threshold cycle values to copies per μL of template. Limit of detection was established at 500 copies.

**Table 1:** Primer sequences used for amplification of the P3 gene of bacteriophage PR772 via qPCR.

Primer	Sequence	Amplicon size (bp)	Reference
P3 Forward	5′-CCCATTAAGTACGGCGATGTTATG-3′	102	(38)
P3 Reverse	5′-GGCAAGCGGAACCCAATAG-3′		

### Statistical analysis

SPSS version 29 was used for data analysis. Statistical significance was assessed using a two-sided Wilcoxon Signed Rank test for related samples, i.e., to assess surface and mask contamination occurring during hand drying; and using a two-sided Mann–Whitney U test for independent samples, i.e., to compare samples between hand drying methods. Both tests were assessed using a 95% confidence interval; *p* ≤ 0.05 was considered statistically significant.

## Results

Overall, nine hand drying assays were performed using a food dye and either the Dyson A9KJ hand dryer, the Dyson AW+D wall hand dryer or PT, to determine the potential for droplet dispersion in the room environment via splattering and aerosolization of particles.

Sixty hand drying assays were also performed using a bacteriophage solution to determine the potential for microbial contamination of hands, surfaces and facemasks using either Dyson A9KJ hand dryer, Dyson AW+D wall hand dryer or PT, in presence and absence of poor hand washing. Hands contaminated with the bacteriophage solution aimed to represent hands not washed following the recommended guidelines (25), i.e., hands that have not been washed with water and soap for 20s. Each assay involved 2 volunteers: one performing the hand drying and other stationed at 1 m distance.

### User and surface contamination as result of splattering

The potential of each hand drying method to disperse water droplets in the washroom environment, potentially contaminating surfaces, the user, and the bystander was investigated using a food dye solution as visual indicator for contamination. Contamination was measured within predefined areas of the room and of the Tyvek suits/ face shields worn by the volunteers.

The highest level of droplet contamination on the washroom surfaces (floor and wall) was observed in the assays using the A9KJ hand dryer (Table 2). Contamination of the floor area around the hand drying unit/user was on average 3 times higher with A9KJ hand dryer compared with AW+D wall hand dryer and 14 times higher compared with PT. At 1 m from the drying station, droplet levels observed following A9KJ hand dryer use were similar to those observed using the AW+D wall hand dryer, but >8 times higher than following PT use. Contamination on the wall next to the drying station was 78 times higher with A9KJ and 19 times higher using the AW+D wall hand dryer, compared with PT. Wall contamination following PT use was associated with liquid displacement from hands when removing PT from the holder unit, whereas floor contamination resulted of droplet dripping during the hand drying movements. Droplets traveled longer distances following A9KJ hand dryer use, up to 90 cm in a straight line and up to 55 cm in an angle (Figure 1).

**Table 2:** Droplet dispersion observed following hand drying assays performed using food dye.

Surface area (cm x cm)	Paper towels	AW+D wall hand dryer	Airblade 9KJ
Wall (65 × 40)	7 ± 1	139 ± 87	536 ± 277
Floor – immediately underneath the hand dryer (65 × 40)	32 ± 5	158 ± 34	466 ± 277
Floor – 1 m behind hand dryer (65 × 40)	13 ± 6	135 ± 32	104 ± 13
Floor – 1 m at a 45-degree angle from hand dryer (65 × 40)	5 ± 5	109 ± 29	93 ± 33
Hand drying person (cm x cm)			
Face shield (32 × 22)	0	56 ± 30	7 ± 4
Torso (22 × 16)	8 ± 5	288* ± 65	46 ± 25
Leg (22 × 16)	3 ± 2	35 ± 8	61 ± 31
Standby user (cm x cm)			
Face shield (32 × 22)	0	62 ± 4	0
Torso (22 × 16)	0	60 ± 22	1 ± 0.8
Leg (22 × 16)	0	56 ± 30	0

Contamination of surfaces, the individual performing hand drying and a standby user stationed a 1 m distance were investigated with three different hand drying methods. Data shown is the average of three hand drying experiments and standard deviation. *Average of 2 assays, as in the third the spots were too many to count.

However, the user and standby user contamination was higher on the assays performed with AW+D wall hand dryer, for nearly all areas of the Tyvek suit analyzed, particularly on the torso of the hand dryer user (Table 2; Supplementary Figure 2). Although the A9KJ hand dryer was associated with high floor contamination at 1 m from the drying unit, almost no droplets were observed on the standby user.

Overall, PT was the method associated with lower person contamination, with no droplets observed in the Tyvek suit or visor of the standby user, and under 10 droplets observed on the hand drying volunteers’ suit (Supplementary Figure 3). Surface contamination was also lower with PT, compared with the other methods, with most droplets observed on the wall traveling up to 15 cm (Figure 1).

### Contamination of surfaces and N95 masks by microbial droplets

Hand drying assays were also performed following hand immersion in a bacteriophage solution, to assess the efficacy of each method in supporting hand hygiene of poorly washed hands. Viral contamination as result of splattering was investigated by recovering N95 respirators and sampling disposable plastic aprons worn by volunteers and by standby users during hand drying.

**Figure 2:**
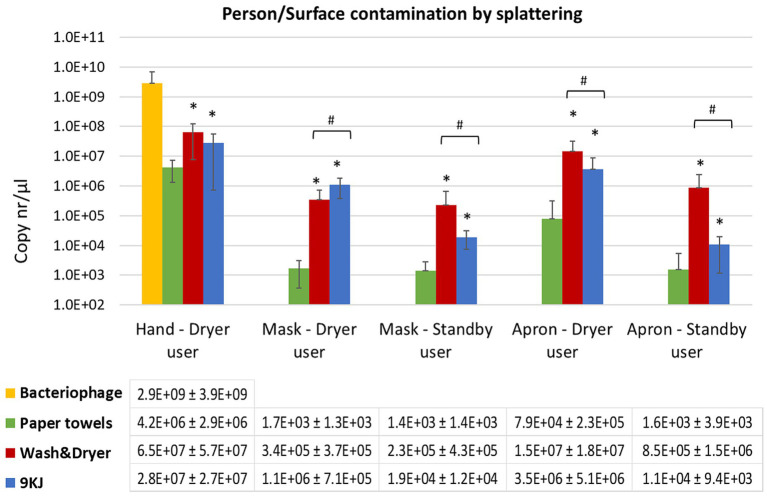
Mean qPCR bacteriophage levels recovered from facemasks and torso of the individual performing the hand drying and the standby user at 1 m distance. Data shown is the average of 10 hand drying experiments with each method and standard deviation. *p < 0.05 significant differences between paper towels and Dyson AW+D wall hand dryer, and between PT and Dyson A9KJ hand dryer, using a two-tail Mann–Whitney U test. ^#^*p* < 0.05 significant differences between Dyson AW+D wall hand dryer and Dyson A9KJ hand dryer using a two-tail Mann–Whitney U test.

Hand contamination following hand drying significantly declined with all methods tested (*p* < 0.05 on the Wilcoxon test), when compared to the stock solution used to contaminate the hands. However, hand contamination following drying was 10-fold lower using PT, when compared to the use of either electric hand dryer (Figure 2). This lower microbial load was associated with a significantly lower 100-fold contamination of the door handle when PT were used, compared to the AW+D wall hand dryer and A9KJ hand dryer (*p* < 0.05 using a two-tailed Mann–Whitney test; Supplementary Figure 4). Among all methods, the bacteriophage transfer from hands to the door handle, was significantly higher following the use of the AW+D wall hand dryer, compared to the A9KJ hand dryer and PT.

Mask contamination of the volunteers performing the hand drying of poorly washed hands was 200-fold and 1,000-fold lower following PT use (1.7 ×10^3^ copies/μl) compared to the use of the AW+D wall hand dryer (3.4 ×10^5^ copies/μl) and the A9KJ hand dryer (1.1 ×10^6^ copies/μl), respectively (Figure 2). Mask contamination above the limit of detection in this position was observed in 80% of the PT assays, whereas electric hand dryers resulted in 100% of tests positive for bacteriophage presence in facemasks.

Similarly, mask contamination of participants standing at 1 m distance of the hand drying unit was 10-fold and 100-fold lower in assays using PT, when compared to the use of A9KJ hand dryer and AW+D wall hand dryer, respectively. (Figure 2). At this position, facemasks contamination with bacteriophage was observed in 100% of the assays using electric hand dryers, but only in 70% of the assays using PT.

Compared to the electric hand dryers, PT resulted in significantly less splattering contamination of both masks and aprons at both investigated positions. This was particularly evident on the hand drying position where the electric methods were associated with 100- to 1,000-fold more apron contamination (Figure 2). For most surfaces tested, AW+D wall hand dryer resulted in a significantly higher facemask contamination compared to both A9KJ hand dryer and PT. The one exception was observed on the splattering of facemasks used by the volunteers during hand drying, where A9KJ hand dryer was associated with the highest contamination.

### Mask contamination as result of aerosols

After hand drying, the participants remained at their defined positions, i.e., by the hand drying station and at 1 m distance from the hand dryer. Volunteers wore a new N95 respirator for each 5-min period tested: at 10–15 and at 25–30 min post-hand drying. The purpose was to assess if each hand drying method created aerosols that could deposit on the masks surface when subjected to the air displacement associated with regular breathing.

Hand drying using AW+D wall hand dryer and A9KJ hand dryer resulted in mask contamination by aerosols at both positions and at all timepoints, whereas this was observed in only 60% of the testing points when PT were used.

**Figure 3:**
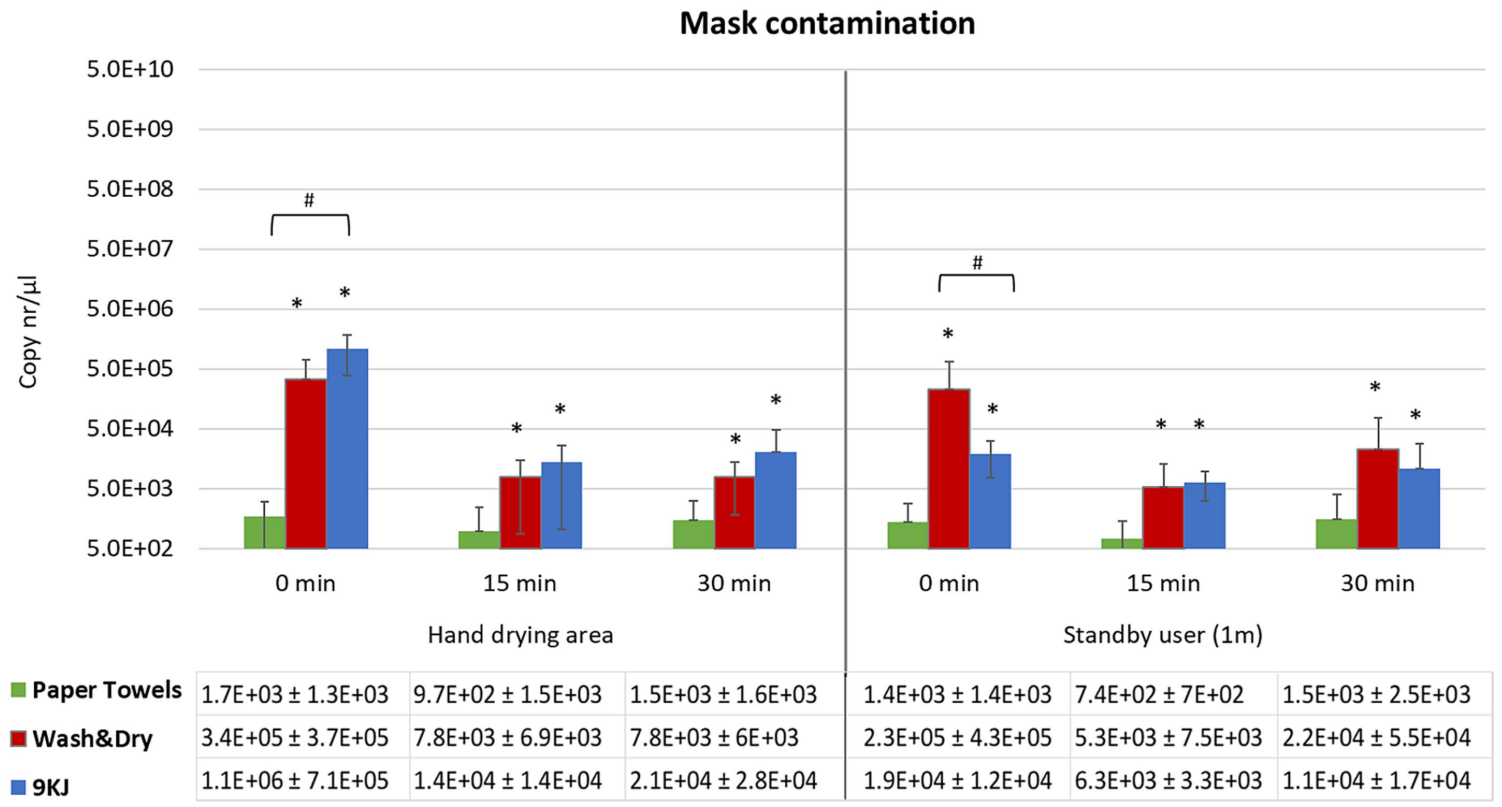
Mean qPCR bacteriophage levels and standard deviation recovered from N95 masks following hand drying. Bacteriophage deposition was investigated at 0 min, 15 min and 30 min post-hand drying, both at the hand drying station and at 1 m distance. Facemasks represented the risk for virus inhalation. *p < 0.05 significant differences between PT and Dyson AW+D wall hand dryer, and between PT and 9KJ, using a two-tail Mann–Whitney U test. ^#^*p* < 0.05 significant differences between Dyson AW+D wall hand dryer and A9KJ hand dryer using a two-tail Mann–Whitney U test.

At the 15-min timepoint, bacteriophage aerosolization at the hand drying station position was 10-fold lower with PT than with the AW+D wall hand dryer, and 100-fold lower compared to the A9KJ hand dryer (Figure 3).

Contamination of facemasks at the hand dryer position was highest at all time points investigated when the A9KJ hand dryer was used. However, at the standby position, facemask contamination was generically higher when AW+D wall hand dryer was used, with exception of the 15 min timepoint, where A9KJ hand dryer resulted in a non-significantly higher mask contamination compared with the AW+D wall hand dryer (Figure 3).

For all methods there was a slight increase on mask contamination observed at 30 min compared to 15 min.

## Discussion

Hand drying is an integral step of hand hygiene (25, 26) and is essential in minimizing the risk of pathogen spread (1, 20, 21, 27). Previous studies reported different results regarding the efficacy of electric hand dryers in removing microbial contamination from individuals’ hands and their potential for contamination of the washroom environment (1, 18, 19, 28, 29). Those observations are likely impacted by the different experimental designs, with a hand washing step of 20s following WHO guidelines (25) and/or hand drying performed until all moisture has been removed, often featuring in experiments where electric hand dryers performed best. However, in real-life settings, poor hand wash practices (no soap, less than 20s) and hand drying for short periods are common (29–32). One study comparing different hand drying methods found that 68% of their volunteers used a jet air dryer for up to 10s in their daily life, whereas experiments employed an average time of >27s to achieve full hand dryness (29). This is relevant as residual moisture in hands can increase microorganism transfer from hands to surfaces (1, 27).

We looked to investigate the efficacy of new electric hand drying systems, the Dyson AW+D wall hand dryer and the A9KJ hand dryer, in removing contamination from hands and their potential to cause particle aerosolization for an extended period, compared with PT.

PT resulted in significantly lower splattering contamination of all surfaces, which was associated with lower transfer of microbial contamination to the door handle via direct contact, similar to previous reports (1).

Droplet dispersion of both the food dye and bacteriophage assays showed A9KJ hand dryer was associated with a higher contamination of walls and floor, whereas person contamination was higher when using the Dyson AW+D wall hand dryer. Both hand dryers have a 2-arm design and similar air-drying velocity; however, the 30° hand drying angle in A9KJ appears to displace more air toward the adjacent surfaces (wall and floor), whereas the horizontal movement of hands during AW+D use suggests more air is displaced towards the nearby individuals.

Facemask contamination via aerosols was significantly lower at all time points and distances investigated, following hand drying with PT, compared with using the Dyson AW+D wall hand dryer or the A9KJ hand dryer. However, bacteriophage deposition on facemasks increased at 30 min post-hand drying with all methods versus the 15 min timepoint, but this increase was 10-fold higher with AW+D wall hand dryer and A9KJ hand dryer, at the standby user position. Hand drying can result in aerosolization of different size particles, with those smaller than 0.5 mm not visible by the naked eye (33). Our data shows that AW+D and A9KJ result in a larger amount of droplets forming and dispersing in the environment compared to PT. Based on these observations, and reported data showing bacteria aerosols ranging from 0.3 μM to 5 μM are formed during hand drying (28), we hypothise that larger and smaller size particles containing bacteriophage are also formed that remain airborne for different periods of time. This appears to be consistent with the different size of bacterial aerosols collected using an air sampler, following hand washing and drying with different methods (28). Similar to many public spaces, the room used in our study has standard ventilation and hand drying assays were performed in sets of 5, to represent repeated use of public washrooms. The air displacement caused by repeated hand drying would support the smaller size viral particles remaining airborne for longer and traveling from the hand drying station to the standby user position at 1 m distance, where the largest increase in mask contamination was observed at 30 min post-hand drying. These results are similar also to those observed when testing the Dyson Airblade jet hand dryer (1, 20–22) and would mean a potential for prolonged exposure of others to microbial pathogens.

Overall, in this study PT use resulted in lower hand, person, and surface contamination, compared with use of AW+D wall hand dryer and A9KJ hand dryer. However, our observations are limited by the room dimension and ventilation and therefore cannot represent every real-world setting. Different hand dryer models and variations in installation heights/angles could also impact droplet dispersion and aerosolization in public spaces.

Studies looking at hand washing practices in the community have reported rates of hand washing with soap in adults as low as 11% (32), whereas a systematic review looking at global hand washing practices in the context of diarrheal diseases has estimated that only 19% of the world population washes their hands with soap after contact with excreta (30). Even when regular hand wash is performed with plain or antimicrobial soap, 4 to 6 log_10_ colony forming units of bacteria have been reported to remain in hands (34–36). Considering how often hand washing is suboptimal, effective hand drying is essential to aid removal of microbes remaining on hands.

## Conclusion

This study investigated person, surface and mask contamination during and up to 30 min after three hand drying methods. Microbial contamination was significantly lower when using PT compared with electric hand dryers. Although the use of facemasks to protect from SARS-CoV-2 is no longer common in the community, facemasks are still used in healthcare facilities and by at risk groups, particularly as viral respiratory infections remain high (7, 9, 11, 37). As poorly washed hands in become increasingly common once again (3, 4, 29–32), hand drying can act as supportive measure to reduce the viral transmission in enclosed environments.

Our results demonstrate that hand drying with PT is associated with a lower risk of droplet and aerosol dispersal compared with use of electric hand dryers, particularly when hands are sub-optimally cleaned.

## Data Availability

The raw data supporting the conclusions of this article will be made available by the authors, upon reasonable request.

## Footnotes

^1^ https://www.benchmarksixsigma.com/calculators/sample-size-calculator-for-mann-whitney-test/
