# Correction: Understanding the impact of different hand drying methods on viral aerosols formation and surface contamination in indoor environments

**DOI:** 10.3389/fpubh.2025.1731810

**Published:** 2025-11-06

**Authors:** Frontiers Production Office

**Affiliations:** Frontiers Media SA, Lausanne, Switzerland

**Keywords:** hand drying, paper towels, electric hand dryer, viral contamination, aerosols

The **Conflict of interest** statement was erroneously given as:

“The authors declare that the research was conducted in the absence of any commercial or financial relationships that could be construed as a potential conflict of interest.”

The correct conflict of interest statement is:

“MW has received consulting fees from Astra Zeneca, BioInteractions, Centauri, Debiopharm, Ferring, GSK, Nestlé, Paion, Pfizer, Phico Therapeutics, The European Tissue Symposium, Tillotts.

The remaining authors declare that the research was conducted in the absence of any commercial or financial relationships that could be construed as a potential conflict of interest.”

The original version of this article has been updated.

