# Supplementary figures and images for: Understanding the impact of different hand drying methods on viral aerosols formation and surface contamination in indoor environments
Source: Front Public Health. 2025 Oct 22;13:1664322. doi: 10.3389/fpubh.2025.1664322 (PMC12586034; doi:10.3389/fpubh.2025.1664322)

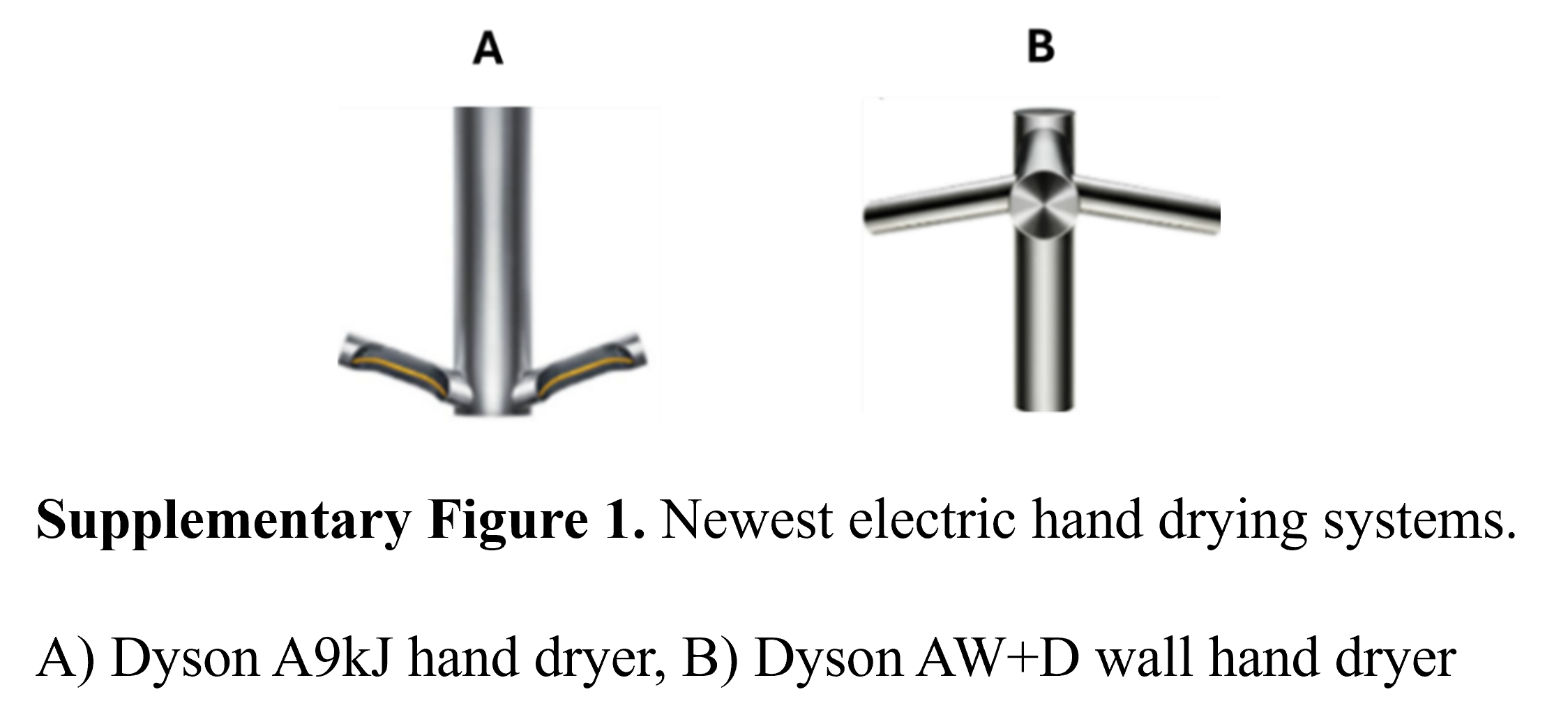

Supplement: Supplementary file 1 [file Image_1.png]

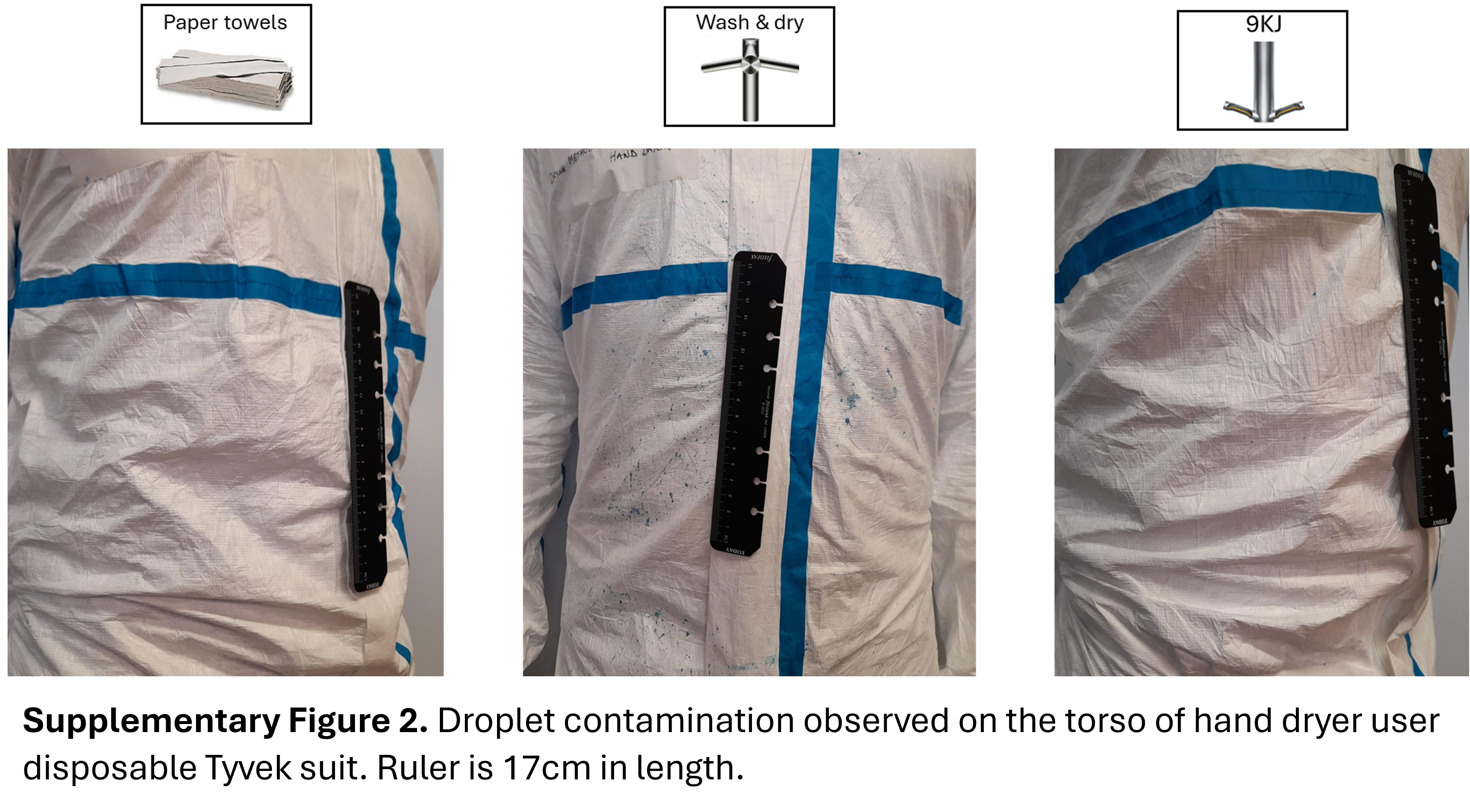

Supplement: Supplementary file 2 [file Image_2.png]

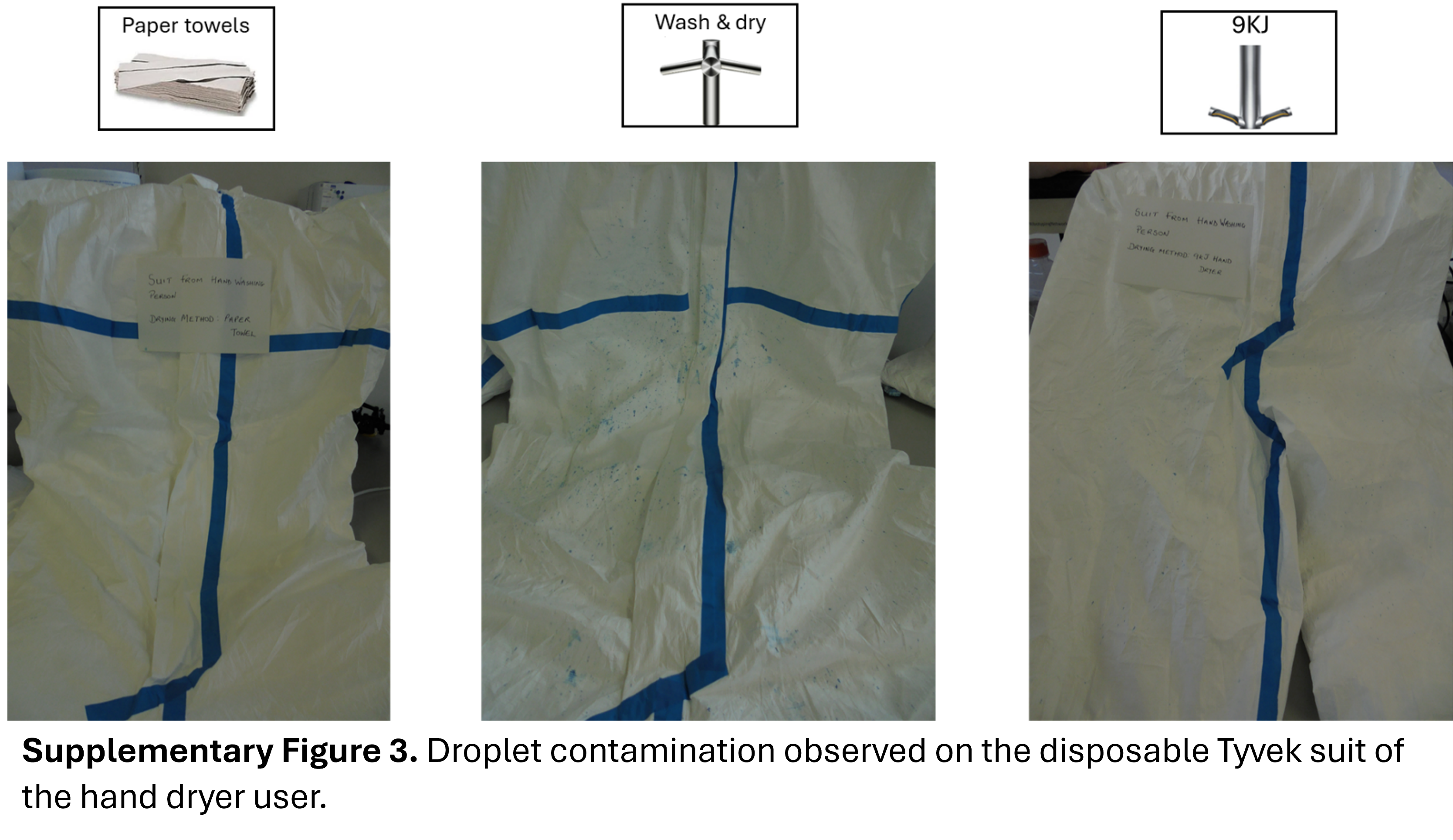

Supplement: Supplementary file 3 [file Image_3.PNG]

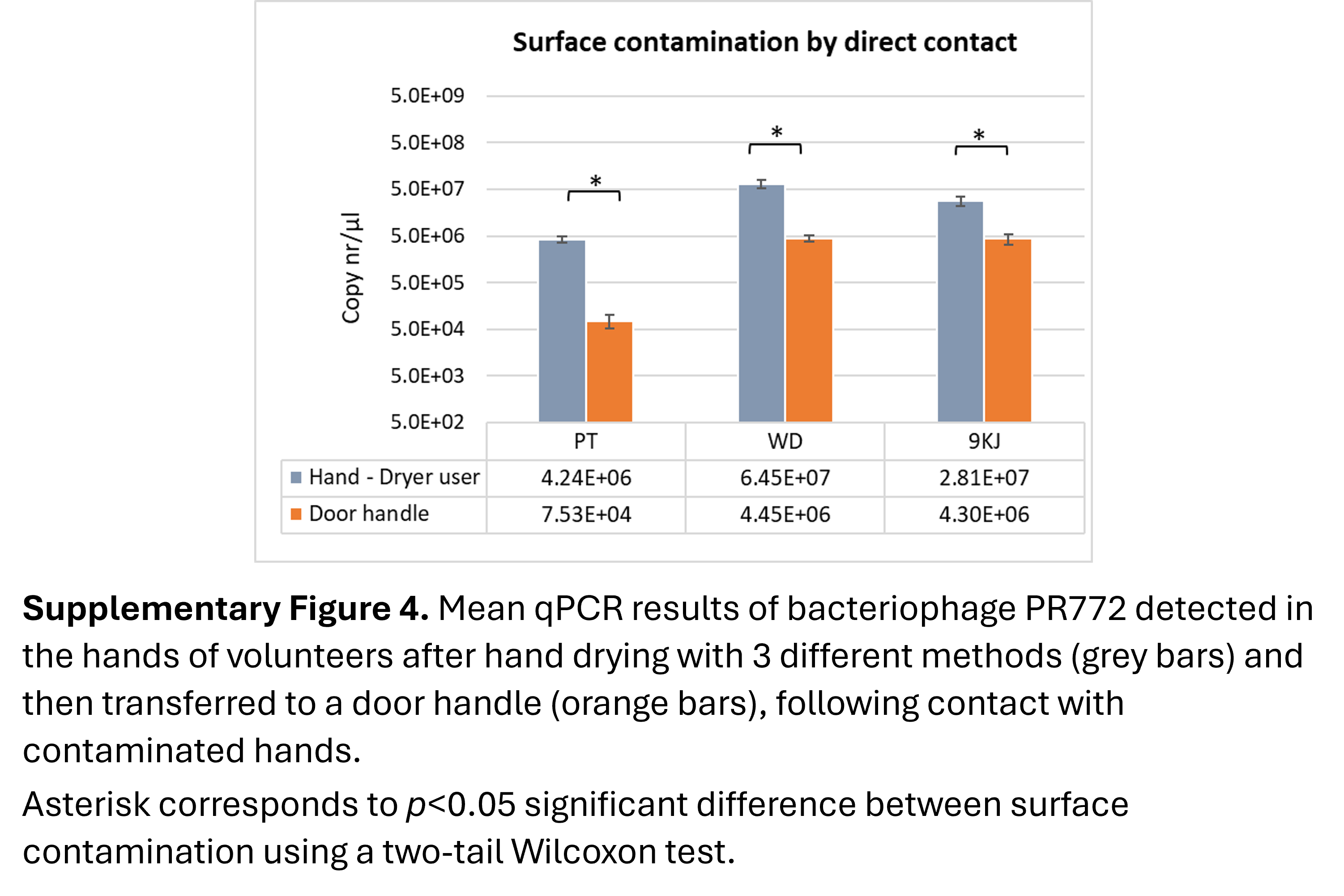

Supplement: Supplementary file 4 [file Image_4.PNG]
